# CTS™ immune cell SR for serum free culture and expansion of human T cells

**DOI:** 10.1186/2051-1426-3-S2-P1

**Published:** 2015-11-04

**Authors:** Grethe Okern, Angel Varela-Rohena, Sandra Kuligowski, Brian Newsom, Tanja Aarvak

**Affiliations:** 1 Thermo Fisher Scientific, Oslo Norway; 2 Thermo Fisher Scientific, Grand Island, NY, USA; 3 Thermo Fisher Scientific, Frederick, MD, USA

## Background

The manufacture of a majority of clinical T cell products for immunotherapy applications requires *in vitro* T cell culture and expansion. Commercialization of T cell manufacturing processes requires reagents that meet regulatory guidelines and ultimately help reduce manufacturing cost of goods. A key component in many T cell culture protocols is human serum, which is expensive and requires extensive testing prior to use for the manufacture of cGMP-compliant T cell therapies. To this end, we have developed a xeno-free serum replacement, CTS™ Immune Cell SR, with defined components that can be used in combination with multiple cell culture media to support *in vitro* expansion of functionally intact T cells.

## Results

T cells activated and expanded with Dynabeads^®^ CD3/CD28 CTS^TM^ and cultured in CTS™ OpTmizer^™^ T cell Expansion SFM, X-Vivo^TM^ 15, or CTS™AIM-V^®^ supplemented with pooled human serum or serum free CTS™ Immune Cell SR showed similar growth kinetics, total fold expansion and transduction efficiency after 2 weeks in culture. Numbers of CD4^+^ and CD8^+^ T cells were comparable in cultures expanded with media containing human serum or CTS™ Immune Cell SR. T cells demonstrated efficacy when infused in an *in vivo* leukemia mouse model. T cell engraftment and leukemia control were similar between mice treated with T cells grown in media containing human serum or CTS™ Immune Cell SR.

## Conclusions

These studies demonstrate that human serum may be replaced by a xeno-free formulation in combination with several commonly used T cell culture media to support *in vitro* expansion and lentiviral transduction of polyclonal T cells. Culturing T cells in CTS™ Immune Cell SR facilitates a favorable culture profile and immune function. Serum free CTS™ Immune Cell SR contains only fully tested human-derived or human recombinant proteins which facilitates supply security for large-scale production of clinical and commercial therapies.

